# The Recalled Age of Initiation of Multiple Tobacco Products among 26–34 Year Olds: Findings from the Population Assessment of Tobacco and Health (PATH) Study Wave 1 (2013–2014)

**DOI:** 10.3390/ijerph17239000

**Published:** 2020-12-03

**Authors:** Adriana Pérez, Elena Penedo, Meagan A. Bluestein, Baojiang Chen, Cheryl L. Perry, Melissa B. Harrell

**Affiliations:** 1 Department of Biostatistics and Data Science, School of Public Health, The University of Texas Health Science Center at Houston (UTHealth), Austin, TX 78701, USA; Baojiang.Chen@uth.tmc.edu; 2 Michael & Susan Dell Center for Healthy Living, School of Public Health, The University of Texas Health Science Center at Houston (UTHealth), Austin, TX 78701, USA; Elena.Penedo@uth.tmc.edu (E.P.); Meagan.A.Bluestein@uth.tmc.edu (M.A.B.); Cheryl.L.Perry@uth.tmc.edu (C.L.P.); Melissa.B.Harrell@uth.tmc.edu (M.B.H.); 3 Department of Health Promotion and Behavioral Sciences, School of Public Health, The University of Texas Health Science Center at Houston (UTHealth), Austin, TX 78701, USA; 4 Department of Epidemiology, Human Genetics and Environmental Sciences, School of Public Health, The University of Texas Health Science Center at Houston (UTHealth), Austin, TX 78701, USA

**Keywords:** cigarettes, cigarillos, traditional cigars, filtered cigars, hookah, smokeless tobacco, e-cigarettes, nationally representative, histogram, hazard ratio

## Abstract

This study examined the recalled age of initiation of seven different tobacco products (TPs) and explored potential influences of sex, race/ethnicity, and cigarette-smoking status on tobacco use initiation among adults 26–34 years old using the PATH study. Methods: Secondary analyses were conducted in the adult restricted PATH wave 1 (2013–2014) dataset. Weighted statistics are reported using the balanced repeated replication method and Fay’s correction to account for PATH’s complex study design. Distributions and histograms of the recalled age of initiation of seven different TPs (cigarettes, cigarillos, traditional cigars, filtered cigars, hookah, smokeless tobacco, and e-cigarettes) are reported, as well as the impact of sex and race/ethnicity using Cox proportional hazard models. The impact of cigarette-smoking status on the recalled age of initiation of each tobacco product other than cigarettes was explored. Results: The highest modes of the recalled age of initiation of cigarette use were at 14–15 and 15–16 years old. The distributions of the recalled age of initiation of cigarillos, traditional cigars, filtered cigars, hookah, and smokeless tobacco occurred later, with the highest modes at 15–16 and 17–18 years old. The distribution of the recalled age of initiation of e-cigarettes had a different shape than the other TPs, with the highest mode reported at 27–28 years old. Conclusion: Due to the ever-changing tobacco marketplace, understanding when contemporary adults aged 26–34 years recall initiating TP use is important and will inform prevention researchers.

## 1. Introduction

The National Survey of Drug Use and Health (NSDUH) is a cross-sectional annual nationally representative cohort study that provides current information on tobacco, alcohol, and drug use in the United States [1]. Almost 82% of 30–39 years old in the 2010 NSDUH data recalled first trying a cigarette before or by 18 years of age, 14.8% recalled first using a cigarette between 18–24 years old, 3.1% recalled first using a cigarette between 25–30 years old, and 0.6% recalled first using a cigarette between 31–39 years old [2]. Using pooled data from 2002–2012, the NSDUH reported that 86% of adults 26–34 years old recalled initiating cigarette use by age 18 [3]. Additionally, the distribution of the recalled age of initiation depicted those who used 99 or fewer cigarettes and those who used 100+ cigarettes in their lifetime, as 100+ cigarettes is a measure used to identify regular adult cigarette users [1,2,3,4,5,6]. They found that those who had used 100+ cigarettes in their lifetime had earlier ages of initiation, with more initiating at 13 years old, compared to those who had used 99 or fewer cigarettes in their lifetime [3].

While the landmark findings listed above provided crucial guidance to tobacco interventions, it is important to examine age of initiation across multiple nationally representative samples with recent data. Importantly, previous research has found that earlier ages of cigarette initiation are associated with increased nicotine dependence [2,7], fewer quit attempts [8], the transition to daily smoking [9], and increases in the risk of chronic diseases [10,11,12]. Understanding when individuals initiate tobacco products (TPs) will inform policy makers and tailor intervention strategies to people of those specific ages, as well as help to educate the public about when these behaviors emerge. Data from the 2013–2014 Population Assessment of Tobacco and Health (PATH) study, a longitudinal nationally representative study of tobacco use and health, reported the prevalence of cigarette use among people 18–24 years old and ≥25 years old as 28.8% and 21.6%, respectively [13]. Although there is an abundance of research on cigarette initiation, there is a gap in the literature reporting the recalled age of initiation of non-cigarette TPs among adults. While the National Health Interview Survey (NHIS) in 2018 estimated that 49.1 million U.S. adults (19.7%) reported currently using any tobacco product, including cigarettes (13.7%), cigars (3.9%), e-cigarettes (3.2%), smokeless tobacco (2.4%), and pipes/hookah (1.0%) [14], this study did not examine the age of initiation of each tobacco product.

We seek to report the distribution of the recalled age of initiation of seven different TPs (cigarettes, cigarillos, traditional cigars, filtered cigars, hookah, smokeless tobacco, and electronic cigarettes) among U.S. adults 26–34 years old using 2013–2014 PATH wave 1 data. This age group allows for a direct comparison with the previously published NSDUH distribution for the recalled age of initiation of cigarettes [3], which found that 99% of adult daily cigarette users recall initiating before age 26 [2]. In addition, it is important to replicate findings across national samples with contemporary data. We hypothesize that the distribution of the recalled age of cigarette initiation from the NSDUH will be similar to the PATH 2013–2014 distributions for all the TPs, except for e-cigarettes since they were not prevalent until 2014 [15]. This study also examined the potential influences of sex, race/ethnicity, and cigarette-smoking status on the recalled age of tobacco use initiation (i.e. cigarillos, traditional cigars, filtered cigars, hookah, smokeless tobacco, and electronic cigarettes) among adults 26–34 years old using PATH, which has never been explored before.

## 2. Materials and Methods

### 2.1. Study Design

The PATH study used a complex sampling design [16,17,18] to generate a nationally representative sample of non-institutionalized youths and adults in the U.S. in the year 2013 [19]. It is a longitudinal cohort study conducted annually. The PATH researchers used a four-stage stratified area probability sampling design with weighting to account for different probabilities of selection and non-response [19]. The response rate for the 2013–2014 PATH wave 1 adult sample was 74.0% [19]. Further details on the PATH study design are described in the PATH Study Restricted Use Files User Guide [20,21]. We obtained access to the restricted-use versions of the PATH adult dataset for wave 1 (2013–2014) and used the Inter-university Consortium for Political and Social Research (ICPSR) server through the University of Michigan to access the data [20]. We conducted secondary data analyses of the recalled age of initiation of seven different TPs among adults 26–34 years old. IRB approval for this study was obtained from the Committee for the Protection of Human Subjects at the University of Texas Health Science Center at Houston with number HSC-SPH-17-0368.

### 2.2. Participants and Measures

**Figure 1:**
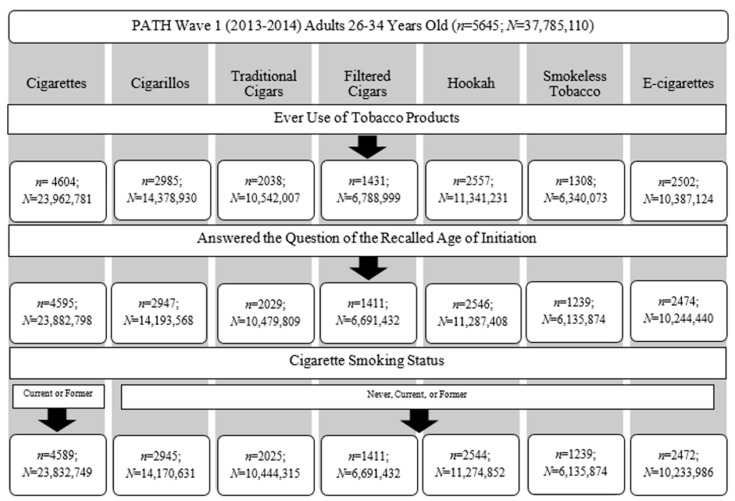
Flowchart depicting how the final sample sizes for each tobacco product (TP) were obtained for analyses. PATH: Population Assessment of Tobacco and Health.

The 2013–2014 PATH wave 1 sample contained 32,320 adult participants (representing *N* = 236,691,585) and the dataset was limited to 5645 (*N* = 37,785,110) participants who were 26–34 years old and were potentially eligible to include in analyses. Only users of each tobacco product who reported their recalled age of initiation were included in the analysis for that tobacco product. Figure 1 shows the application of inclusion criteria to obtain the final sample sizes for the analysis of each of the seven TPs.

### 2.3. Tobacco Product Use

PATH used seven questions to determine ever use of each tobacco product: (i) “Has ever smoked a cigarette even one or two puffs”, (ii) “Has ever smoked a cigarillo even once or twice”, (iii) “Has ever smoked a traditional cigar, even once or twice”, (iv) “Has ever smoked a filtered cigar, even once or twice”, (v) “Has ever smoked a hookah, even one or two puffs”, (vi) “Has ever used smokeless tobacco, even once or twice”, and (vii) “Has ever used an e-cigarette even once or twice”. Response options to all these questions included “yes”, “no”, “don’t know”, and “refused”. Participants who answered “yes” to the previous questions were classified as ever users of that product. Participants who answered “don’t know” and “refused” were considered missing and were excluded from analysis, as shown in Figure 1. The percent of missingness remained low but varied across the TPs from 0.1–3.1%. It is worth noting that cigarillo use does not include marijuana, as PATH has another variable for cigarillo with marijuana use that was not used here.

### 2.4. Lifetime Use of Cigarettes

PATH used a survey question that asked participants: “How many cigarettes have you smoked in your entire life? A pack usually has 20 cigarettes in it”. Response options included: “1 or more puffs but never a whole cigarette”, “1 to 10 cigarettes (about 1/2 pack total)”, “11 to 20 cigarettes (about 1/2 pack to 1 pack)”, “21 to 50 cigarettes (more than 1 pack but less than 3 packs)”, “51 to 99 (more than 2 1/2 packs but less than 5 packs)”, and “At least 100 or more cigarettes (5 packs or more)”. Participants were collapsed as either having smoked 100+ cigarettes in their lifetime or 99 or fewer cigarettes in their lifetime.

### 2.5. Cigarette Smoking Status

To further understand the recalled age of initiation of the six TPs other than cigarettes, we explored differences in the recalled age of initiation by cigarette smoking status, which classified participants as never, current, or former cigarette users. Never cigarette users were those who answered “no” to the survey question about ever cigarette use. Ever cigarette users were further classified as current users if they reported any cigarette use in the past 30 days. We used two derived variables created by PATH to classify participants as former cigarette users: (i) former established cigarette users were participants who answered that they have ever smoked a cigarette, have smoked more than 100 cigarettes in lifetime, and now do not smoke at all; and (ii) former experimental cigarette users were participants who answered that they have ever smoked a cigarette, have not smoked more than 100 cigarettes in lifetime, and now do not smoke at all. These variables were combined into a single measure to represent former cigarette use. Participants who did not provide answers to these questions needed to categorize them as either current or former cigarette users were excluded from analysis, as shown in Figure 1.

### 2.6. Recalled Age of Initiation

We identified participants who provided an answer for the questions on to the recalled age of initiation of the seven different TPs, which asked: (i) “How old were you the first time you smoked part or all of a cigarette?”, (ii) “How old were you the first time you smoked part or all of a filtered cigar, even one or two puffs?”, (iii) “How old were you the first time you smoked part or all of a traditional cigar, even one or two puffs?”, (iv) “How old were you the first time you smoked part or all of a cigarillo, even one or two puffs?”, (v) “How old were you the first time you smoked hookah, even one or two puffs?”, (vi) “How old were you when you first used smokeless tobacco, even one or two times?” and (vii) “How old were you when you first used an e-cigarette, even one or two times?”. Participants submitted a numeric answer to these questions to represent their recalled age of initiation. Participants who answered the question on the recalled age of initiation were included in analysis for that tobacco product, as shown in Figure 1.

### 2.7. Sociodemographic Characteristics

PATH used an imputed variable for sex which categorized participants as males and females for participants who did not report their sex (less than 1%) [20]. PATH also imputed a variable for participant race (1.0% imputed in the original PATH wave 1 sample), which included the following categories to measure participant race: white race alone, black race alone, Asian race alone, and other race (including multi-racial). PATH also used an imputed variable (less than 1.0% imputed in the original PATH wave 1 sample) to measure ethnicity of participants as either Hispanic or non-Hispanic. We collapsed these two variables into a race/ethnicity variable to be comparable with previous publications of the Surgeon General’s report [2]. Those who answered non-Hispanic ethnicity and white race were categorized as “Non-Hispanic white”, non-Hispanic ethnicity and black race alone were categorized as “Non-Hispanic black”, Hispanic ethnicity as “Hispanic”, and non-Hispanic other (non-Hispanic Asian, multi-race, and other races) categorized as “Non-Hispanic other”. Details on education level and household income are provided to give a description of our analytic sample. The highest level of education obtained was asked for with the following question: “What is the highest grade or level of school you completed?”. Response options included: “less than high school”, “some high school, no diploma”, “General Education Development (GED)”, “high school graduate—diploma”, “some college but no degree”, “Associate degree—occupational/vocational”, “Associate degree—academic program”, “bachelor’s degree (ex: BA, AB, BS)”, “master’s degree (ex: MA, MS MEng, Med, MSW)”, “professional school degree (ex: MD, DDS, DVM, JD)”, and “doctorate degree (ex: PhD, EdD)”. Due to small sample size, response categories were collapsed as follows: “less than high school/GED”, “high school graduate”, “some college/associate degree”, “bachelor’s degree” and “advanced degree”. Total household income in the past 12 months was asked for with the following question: “Which of the following categories best describes your total household income in the past 12 months?”. Response categories included: “less than USD 10,000”, “USD 10,000–14,999”, “USD 15,000–24,999”, “USD 25,000–34,999”, “USD 35,000–49,999”, “USD 50,000–74,999”, “USD 75,000–99,999”, “USD 100,000–149,999”, “USD 150,000–199,999”, and “USD 200,000 or more”. Due to small sample size, response categories were collapsed as follows: “less than USD 10,000”, “USD 10,000–24,999”, “USD 25,000–49,999”, “USD 50,000–74,999”, “USD 75,000–99,999”, “USD 100,000–149,999”, and “USD 150,000 or more”.

### 2.8. Statistical Analysis

Weighted frequencies and percentages are reported for categorical variables and weighted means and standard errors are reported for continuous variables. The distribution of the recalled age of initiation incorporated the cross-sectional wave 1 sampling weights and 100 balanced repeated replicate (BRR) weights with Fay’s correction factor of 0.3 to account for PATH’s complex study design, including stratification and clustering [19]. Histograms and distributions are reported overall for the seven TPs, as well as the stratified analysis of cigarettes among those who used 99 or fewer cigarettes in their lifetime and those who used 100+ cigarettes in their lifetime. These histograms were formulated in order to directly compare the results of the previously published NSDUH histogram depicting the recalled age of cigarette initiation [3]. Due to the small sample size of initiation for each age in years, across each TP, ages were combined in order to produce more stable estimates (e.g., 5–10, 12–15, 14–15, etc.). The way that ages were combined for each tobacco product is shown in the tables. Differences in the recalled age of initiation of cigarillos, traditional cigars, filtered cigars, hookah, smokeless tobacco, and e-cigarettes were explored by sex, by race/ethnicity, and by cigarette smoking status (never, current, or former) using weighted Cox proportional hazard models. Hazard ratios (HRs) and 95% confidence intervals (CIs) are reported. All statistical analyses were completed using SAS version 9.4.

## 3. Results

Table 1 reports sociodemographic characteristics by use of each one of the seven TPs. Among the 26–34 year old ever users of TPs who reported their recalled age of initiation, there was an estimated national total of 23.8 million cigarette users, 14.1 million users of cigarillos, 11.2 million users of hookah, 10.4 million users of traditional cigars, 10.2 million e-cigarette users, 6.7 million users of filtered cigars, and 6.1 million users of smokeless tobacco, in 2013–2014. Across all seven TPs, males report higher proportions of tobacco product use in comparison to women; Non-Hispanic whites are using more of these TPs compared to any other race; the majority of ever users also reported an education level below a bachelor’s degree; the majority of ever users reported household income less than USD 50,000; and most ever users reported a high proportion of current cigarette use across the other six TPs. Among cigarette users, their mean age was 30.1 (SE = 0.05), 54.2% were male, 63.9% were Non-Hispanic white, 33.7% had some college/associate degree, and 26.5% had an annual household income of USD 25,000–49,000. Please see Table 1 for the sociodemographic characteristics for the other TP users.

**Figure 2:**
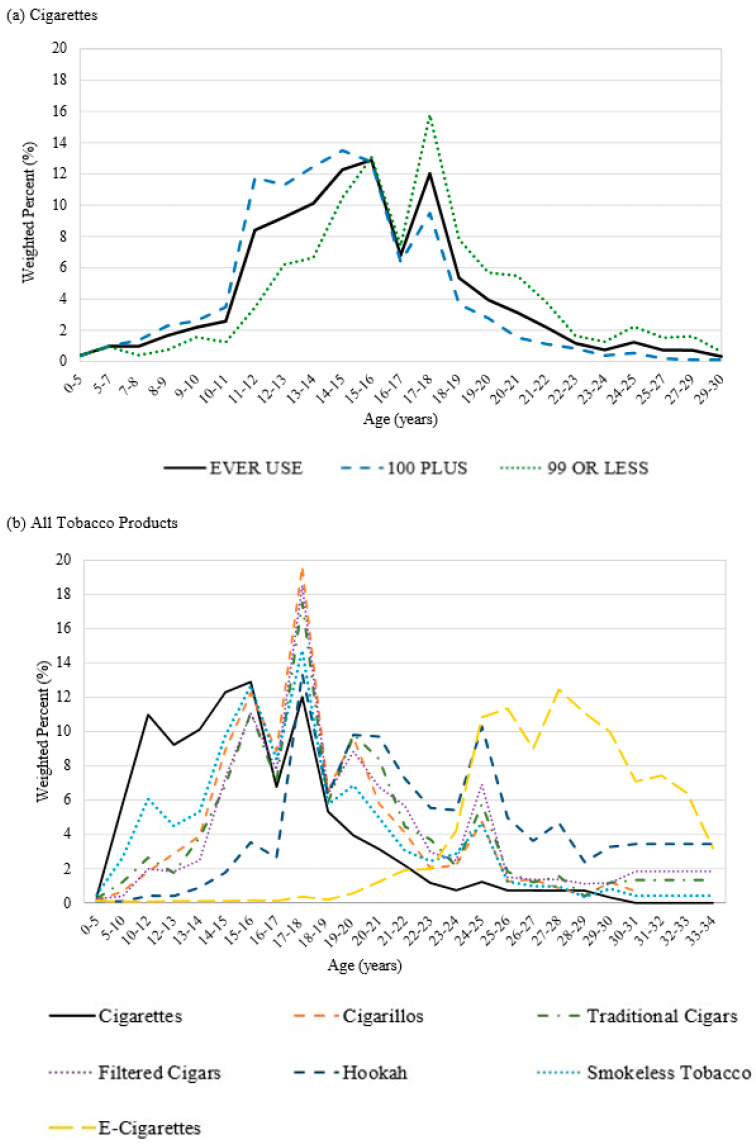
Histograms of the recalled age of initiation of TP use among PATH wave 1 26–34 year olds.

Table 2 presents the weighted percentages for the recalled age of initiation across the seven different TPs, as well as the distribution of recalled age of cigarette initiation stratified by lifetime cigarette use. Figure 2 displays the histograms with the recalled age of initiation of each tobacco product and the cigarette distribution stratified by lifetime cigarette use. The distribution of the recalled age of initiation for those that have used 100+ cigarettes in their lifetime has modes at 13–14 (12.47%), 14–15 (13.5%), and 15–16 (12.78%). The distribution of the recalled age of initiation for those that used 99 or fewer cigarettes in their lifetime has modes of initiation at later ages, including 14–15 (10.47%), 15–16 (13.06%), and 17–18 (15.77%). Across the different TPs, cigarillos, traditional cigars, filtered cigars, and smokeless tobacco show similar bimodal distributions of the recalled age of initiation occurring at 15–16 (12.26%, 11.13%, 11.08%, 12.68%, respectively) and 17–18 (19.64%, 17.52%, 18.52%, 14.74%, respectively) years old. On the contrary, the overall distribution for cigarette users shows a shape with three modes of the recalled age of initiation occurring at 14–15 (12.28%), 15–16 (12.89%), and 17–18 (12.03%) years old. Hookah users exhibited the highest mode of recalled age of initiation at 17–18 (13.37%) years old, but this distribution was shaped differently from the others with another mode occurring at 24–25 (10.3%) years old. In contrast, the distribution for the recalled age of e-cigarette initiation was different from the other TPs with three modes of initiation occurring at 25–26 (11.36%), 27–28 (12.45%), and 28–29 (11.1%) years old.

Table 3 presents the results examining differences in the recalled age of initiation by sex, by race/ethnicity, and by cigarette-smoking status for the six TPs other than cigarettes. In comparison to females, males had increased risk of recalling initiating TP use at earlier ages for cigarillos (HR = 1.08; 95% CI = 1.01–1.16), traditional cigars (HR = 1.46; 95% CI = 1.27–1.47), filtered cigars (HR = 1.18; 95% CI= 1.05–1.32), hookah (HR = 1.10; 95% CI= 1.02–1.19), and e-cigarettes (1.11; 95% CI= 1.03–1.20). Our analysis revealed that there was no difference in the recalled age of initiation of smokeless tobacco between males and females.

Race/ethnicity groups exhibited differential patterns in the recalled age of initiation across the six TPs. Non-Hispanic blacks had a lower risk of an earlier recalled age of initiation compared to Non-Hispanic whites, with a 26% decrease in the risk of initiating filtered cigars, a 46% decrease in the risk of initiating hookah, and a 31% decrease in the risk of initiating smokeless tobacco, indicating they recalled initiating at later ages compared to Non-Hispanic whites. Hispanics had lower risk of an earlier recalled age of initiation compared to Non-Hispanic whites, with a 15% decrease in the risk of initiating cigarillos, a 23% decrease in the risk of initiating traditional cigars, a 31% decrease in the risk of initiating hookah, and a 37% decrease in the risk of initiating smokeless tobacco, indicating they recalled initiating at later ages compared to Non-Hispanic whites. The Non-Hispanic other race/ethnicity group had a lower risk of an earlier recalled age of initiation compared to Non-Hispanic whites, with a 22% decrease in the risk of initiating cigarillos, a 28% decrease in the risk of initiating traditional cigars, and a 19% decrease in the risk of initiating smokeless tobacco, indicating they recalled initiating at later ages compared to Non-Hispanic whites. There were no differences in the recalled age of e-cigarette initiation by race/ethnicity.

Cigarette-smoking status exhibited different relationships with the recalled age of initiation across the six TPs. In comparison to never users of cigarettes, current cigarette users exhibited increased risk in reporting earlier recalled ages of initiation, with a 21% increased risk of initiating cigarillos and a 59% increased risk of initiating traditional cigars at earlier ages than never cigarette users. Similarly, in comparison to never users of cigarettes, former cigarette users exhibited increased risk in reporting earlier recalled ages of initiation, with a 27% increased risk of initiating traditional cigars and a 20% increased risk of initiating hookah at earlier ages than never cigarette users. There were no significant differences in the recalled age of e-cigarette initiation or age of smokeless tobacco initiation among current or former cigarette users compared to never cigarette users.

## 4. Discussion

This study reports contemporary data on the recalled age of initiation of cigarettes and six other TPs among 26–34 year olds in the 2013–2014 wave 1 PATH study. Before the PATH study was conducted, the NSDUH and the Surgeon General’s report provided the recalled age of initiation of cigarettes among 26–34 year olds [3] and 30–39 year olds [5], respectively. With the changes in the tobacco product marketplace with e-cigarettes entering the market in 2007 [22] and other products providing diversified options, asking adults to recall their age of initiation of other TPs will help provide an understanding of tobacco product use that is not limited to cigarettes. This paper uses participant recall data, which is an efficient way to report the distribution of the recalled age of initiation, as researchers have done before with the recalled age of initiation of cigarettes. Similar to the NSDUH data from 2002–2012 [3], we found in 2013–2014 those who progressed to using 100+ cigarettes in their lifetime reported earlier recalled ages of initiation compared to those who reported using 99 or fewer cigarettes in their lifetime. In addition, both our data and the NSDUH data indicate the highest mode of cigarette initiation occurs at 14–15 years old for those who progressed to using 100+ cigarettes in their lifetime [3]. While our study showed that among the 26–34 year olds who had used 99 or fewer cigarettes in their lifetime, their mode age of initiation occurred between 17–18 years old, the NSDUH found their highest initiation mode one year later at 18–19 years old [3], indicating a possible shift to an earlier age of initiation among the 2013–2014 PATH wave 1 cohort, despite well-established findings that cigarette use is declining [14]. For the overall distribution of ever cigarette use, our data also mirrored the NSDUH data, with both studies finding the highest mode of cigarette initiation occurring around 15 years old [3]. In addition, the Surgeon General’s report provides findings using the 2012 NSDUH among 30–39 year olds, which also found that their highest mode for recalled age of cigarette initiation occurred at 15 years old (15.5%) [5].

Some of the most widely cited findings from the 1994 and 2014 Surgeon General’s report was that 88% and 86.9%, respectively, of adults recalled cigarette initiation occurring by age 18 [5,23]. Approximately 86% of adults 26–34 years old in the 2002–2012 NSDUH recalled initiating cigarettes by age 18 (see Figures 2 and 3 of that report) [3]. In comparison, our study revealed that 80.5% recalled initiating cigarettes by age 18, which may reflect the success of cigarette interventions that were implemented to combat cigarette use in the U.S. [2,5]. To our knowledge, this is the first study to report the full distribution of the recalled age of initiation of six other TPs among adults 26–34 years old. The recalled age of initiation for the other TPs by age 18 was markedly different from the recalled age of initiation for cigarettes. Specifically, our findings also indicated that the recalled age of initiation by age 18 was 59.1% for cigarillos, 52.3% for traditional cigars, 51.6% for filtered cigars, 23.2% for hookah, 54.6% for smokeless tobacco, and 0.96% for e-cigarettes. E-cigarette products are the newest tobacco product on the market, which means that the youngest participants in our study would not have been introduced to these products during early adolescence. It is also known that hookah initiation is common in college students, which could explain the later recalled age of initiation among hookah users [24,25]. We could not find any manuscripts in the tobacco literature to compare with our findings on the recalled age of initiation by sex, race/ethnicity, and cigarette-smoking status. We hope that future researchers will be able to use the findings presented here to determine if there is a consistent age of initiation for each tobacco product.

A different study of PATH waves 1–3 (2013–2016) examined factors associated with the initiation of cigarettes, e-cigarettes, cigars (traditional cigars, cigarillos, and filtered cigars), hookah, and smokeless tobacco among all adults, including 25–39 year olds [13], which is similar to our sample of 26–34 year olds. This study reports incidence rates among never users at baseline, and found that 2.2% initiated cigarettes, 6.8% initiated e-cigarettes, 3.1% initiated cigars, 1.9% initiated hookah, and 0.6% initiated smokeless tobacco over two years of 1-year follow-up periods [13]. In the same study, it was reported that older youth (15–17 year olds) compared to younger youth (12–14 year olds), as well as young adults (18–24 year olds) compared to older adults (25–39 year olds), had increased risk for initiating ever use of all five TPs studied [13]. These findings are reflective of the modes in initiation we found for the recalled age of initiation, which suggest that older adolescence and young adulthood are developmental periods during which the risk of TP initiation is increased, except in hookah and e-cigarette use.

It is imperative to examine TPs other than cigarettes as the new landscape of TPs is quickly changing. In light of the findings presented here, intervention and prevention specialists should target youth with campaigns to prohibit the initiation of all TPs before they turn 18. Tobacco interventions aimed at young adults are typically focused on quitting, yet we found that tobacco initiation does occur in young adulthood (e.g., hookah, e-cigarettes, etc.). Therefore, prevention and intervention specialists should design programs and campaigns to prevent tobacco product initiation not only in adolescents but also in young adults.

The strength of this study is that we used nationally representative data on seven different tobacco products to estimate the recalled age of initiation with contemporary data since the recent changes in the tobacco landscape. The primary limitation of this study was that we relied on participant recall data, but we did so in order to be comparable with the estimates from previous publications [2,3,5]. Additionally, we limited our sample to 26–34 year olds in order to compare with the NSDUH data [3], but this prevented us from comparing our results to other previous studies.

## 5. Conclusions

Understanding the recalled age of initiation in adults is important. However, measuring the age of initiation prospectively would not be affected by recall bias [26], which will be important for future researchers to consider. We found considerable evidence for our hypothesis that the distributions of the recalled age of initiation for cigarettes, cigars, cigarillos, filtered cigars, and smokeless tobacco were similar to the 2002–2012 NSDUH data [3]. We also found evidence for our hypothesis that the distribution for the recalled age of initiation for e-cigarettes would be different since this product was not readily available to our participants during their adolescent development. In addition, contrary to our hypothesis, we found that the distribution for the recalled age of initiation of hookah use was shaped differently than the recalled age of initiation for cigarettes. More research is needed to determine the reasons that the other TPs are initiated at later ages compared to cigarettes. It is important to note that our study sample includes data collected in 2013–2014, before the federal Tobacco 21 law changed the minimum age for the sale of tobacco products to 21 years of age in December 2019 [27]. The data provided in this study can be used to compare with future studies that examine how this law has impacted the age of initiation, which has hopefully prevented individuals of younger ages from accessing tobacco products as well as to educate the public on when these behaviors first occur.

## Figures and Tables

**Table 1:** Sociodemographic characteristics of 26–34 year old adults from PATH ^a^ 2013–2014 (wave 1) who reported ever use of each of the seven tobacco products.

Characteristics	Cigarettes	Cigarillos	Traditional Cigars	Filtered Cigars	Hookah	Smokeless Tobacco	E-Cigarettes
	*n* = 4589;	*n* = 2945;	*n* = 2025;	*n* = 1411;	*n* = 2544;	*n* = 1239;	*n* = 2472;
	*N* = 23,832,749 ^b^	*N* = 14,170,631 ^b^	*N* = 10,444,315 ^b^	*N* = 6,691,432 ^b^	*N* = 11,274,852 ^b^	*N* = 6,135,874 ^b^	*N* = 10,233,986 ^b^
Age ^c^ (SE)	30.1 (0.05)	29.9 (0.06)	30.3 (0.07)	29.9 (0.08)	29.5 (0.06)	30.2 (0.09)	29.8 (0.05)
Sex (%) Male	12,928,562 (54.2)	9,216,543 (65.0)	7,505,658 (71.9)	4,408,176 (65.9)	6,542,032 (58.0)	5,230,834 (85.2)	5,939,161 (58.0)
Race/Ethnicity (%)							
Non-Hispanic White	15,224,497 (63.9)	9,582,486 (67.6)	7,796,819 (74.7)	4,872,992 (72.8)	6,873,686 (61.0)	5,024,445 (81.9)	6,835,995 (66.8)
Non-Hispanic Black	2,598,602 (11.0)	1,886,031 (13.3)	643,557 (6.1)	521,966 (7.8)	1,173,617 (10.4)	198,448 (3.2)	1,085,212 (10.6)
Hispanic	4,377,132 (18.3)	1,884,693 (13.3)	1,358,941 (13.0)	801,952 (12.0)	2,049,958 (18.2)	577,455 (9.4)	1,524,365 (14.9)
Non-Hispanic Other ^d^	1,632,518 (6.8)	817,421 (5.8)	644,998 (6.2)	494,521 (7.4)	1,177,591 (10.4)	335,526 (5.5)	788,413 (7.7)
Education (%)							
<High school/GED	3,929,855 (16.5)	2,175,877 (15.3)	1,218,268 (11.7)	976,045 (14.6)	1,177,211 (10.4)	934,409 (15.2)	1,843,220 (18.0)
High school graduate	4,960,023 (20.8)	2,908,106 (20.5)	1,787,984 (17.1)	1,315,079 (19.7)	1,756,192 (15.6)	1,510,371 (24.6)	2,503,820 (24.5)
Some college/associate degree	8,035,520 (33.7)	5,124,786 (36.2)	3,290,104 (31.5)	2,443,901 (36.5)	3,839,439 (34.0)	1,998,679 (32.6)	3,930,757 (38.4)
Bachelor’s Degree	5,128,006 (21.5)	3,018,755 (21.3)	2,941,730 (28.2)	1,371,621 (20.5)	3,253,487 (28.9)	1,346,746 (22.0)	1,564,097 (15.3)
Advanced Degree	1,779,345 (7.5)	943,107 (6.7)	1,206,229 (11.5)	584,786 (8.7)	1,248,522 (11.1)	345,670 (5.6)	392,092 (3.8)
Household Income (%)							
Less than USD 10,000	3,040,169 (13.6)	1,691,084 (12.6)	849,284 (8.6)	845,562 (13.3)	1,060,945 (9.9)	531,715 (9.2)	1,541,505 (16.1)
USD 10,000–24,999	4,757,818 (21.3)	2,737,539 (20.5)	1,476,571 (15.0)	1,139,093 (18.0)	2,026,093 (18.9)	997,356 (17.1)	1,144,215 (24.1)
USD 25,000–49,999	5,924,051 (26.5)	3,646,654 (27.2)	2,510,408 (25.5)	1,879,872 (29.6)	2,918,611 (27.3)	169,0954 (29.0)	2,842,491 (29.7)
USD 50,000–74,999	3,101,566 (13.8)	1,968,800 (14.7)	1,658,604 (16.8)	910,290 (14.4)	1,720,543 (16.1)	926,452 (16.0)	1,277,120 (13.3)
USD 75,000–99,999	2,660,154 (11.9)	1,587,220 (11.8)	1,455,220 (14.8)	752,543 (11.9)	1,349,708 (12.6)	710,935 (12.2)	822,631 (8.6)
USD 100,000–149,999	2,002,369 (8.9)	1,228,450 (9.2)	1,200,354 (12.2)	529,668 (8.4)	1,022,865 (9.5)	652,521 (11.2)	519,975 (5.4)
USD 150,000+	893,475 (4.0)	540,364 (4.0)	706,228 (7.1)	279,386 (4.4)	612,850 (5.7)	306,554 (5.3)	2,64,955 (2.8)
Missing	1,453,147	770,520	587,645	355,017	563,237	319,385	656,093
Cigarette Smoking Status (%)							
Never	NA	776,385 (5.5)	909,950 (8.7)	224,598 (3.3)	1,271,113 (11.3)	267,710 (4.3)	235,567 (2.3)
Current	11,527,048 (48.4)	7,466,705 (52.7)	4,616,480 (44.2)	3,740,819 (56.0)	5,063,758 (44.9)	3,341,870 (54.5)	7,374,320 (72.1)
Former	12,305,701 (51.6)	5,927,540 (41.8)	4,917,885 (47.1)	2,726,015 (40.7)	4,939,981 (43.8)	2,526,294 (41.2)	2,624,099 (25.6)

^a^ PATH restricted file received disclosure to publish: 26 February 2020. United States Department of Health and Human Services. National Institutes of Health. National Institute on Drug Abuse, and United States Department of Health and Human Services. Food and Drug Administration. Center for Tobacco Products. Population Assessment of Tobacco and Health (PATH) Study (United States) Restricted-Use Files. ICPSR36231-v13.AnnArbor, MI: Inter-university Consortium for Political and Social Research (distributor), 24 June 2020. https://doi.org/10.3886/ICPSR36231.v13. ^b^ N represents the U.S. population size that is estimated from PATH’s complex sampling design, ^c^ weighted mean, ^d^ non-Hispanic other includes Asian, multi-race, etc.

**Table 2:** Weighted percentages of the recalled age of initiation of multiple tobacco products among 26–34-year-old adults 2013–2014 PATH ^a^ (wave 1).

Age Range (Years)	Cigarettes			Cigarillos	Traditional Cigars	Filtered Cigars	Hookah	Smokeless Tobacco	E-Cigarettes
	Ever Use	100+	99 or Fewer	Ever Use					
0–5	0.38	0.36	0.42	0.16	0.27	0.25	0.11	0.48	0.13
5.01–10	5.78	7.23	3.64	0.68	1.22	0.39	0.09	2.66	0.08
10.01–11	2.58	3.48	1.24	1.82	2.65	2	0.42	6.08	
11.01–12	8.41	11.76	3.47						
12.01–13	9.24	11.3	6.2	2.88	1.76	1.83	0.41	4.5	0.11
13.01–14	10.12	12.47	6.65	3.9	3.82	2.49	0.9	5.32	
14.01–15	12.28	13.5	10.47	8.92	6.84	7.27	1.77	9.77	
15.01–16	12.89	12.78	13.06	12.26	11.13	11.08	3.54	12.68	0.15
16.01–17	6.78	6.36	7.41	8.88	7.09	7.78	2.63	8.38	0.12
17.01–18	12.03	9.49	15.77	19.64	17.52	18.52	13.37	14.74	0.37
18.01–19	5.34	3.67	7.81	6.54	5.85	6.36	6.32	5.75	0.2
19.01–20	3.95	2.77	5.69	9.64	9.85	8.84	9.81	6.86	0.57
20.01–21	3.13	1.54	5.47	5.8	8.39	6.77	9.72	4.96	1.24
21.01–22	2.19	1.11	3.79	4.1	4.46	5.67	7.26	3.05	1.92
22.01–23	1.16	0.83	1.64	2.03	3.71	2.96	5.55	2.44	1.98
23.01–24	0.74	0.38	1.27	2.21	2.16	2.47	5.41	2.88	4.23
24.01–25	1.23	0.55	2.23	4.74	5.77	6.93	10.3	4.66	10.84
25.01–26	0.73	0.19	1.52	1.24	1.82	1.49	4.98	1.25	11.36
26.01–27				1.38	1.21	1.37	3.63	0.98	9.02
27.01–28	0.72	0.12	1.62	0.87	1.56	1.38	4.68	0.96	12.45
28.01–29				0.42	0.39	1.12	2.39	0.36	11.1
29.01–30	0.32	0.11	0.63	1.22	1.19	1.18	3.27	0.82	9.98
30.01–31	0	0	0	0.67	1.34	1.85	3.44	0.42	7.1
31.01–32	0	0	0						7.44
32.01–33	0	0	0						6.4
33.01–34	0	0	0						3.21

^a^ PATH restricted file received disclosure to publish: 17 February and 2 March 2020. United States Department of Health and Human Services. National Institutes of Health. National Institute on Drug Abuse, and United States Department of Health and Human Services. Food and Drug Administration. Center for Tobacco Products. Population Assessment of Tobacco and Health (PATH) Study (United States) Restricted-Use Files. ICPSR36231-v13.AnnArbor, MI: Inter-university Consortium for Political and Social Research (distributor), 24 June 2020. https://doi.org/10.3886/ICPSR36231.v13.

**Table 3:** Hazard ratio and 95% confidence interval reported for the recalled age of initiation of tobacco products by sex, race/ethnicity, and cigarette-smoking status among 26–34-year-old adults reported in 2013–2014 PATH ^a^ (wave 1).

Tobacco Product	Cigarillos	Traditional Cigars	Filtered Cigars	Hookah	Smokeless Tobacco	E-Cigarettes
	Hazard Ratio (95% Confidence Interval) ^b^					
Sex						
Female	1.00	1.00	1.00	1.00	1.00	1.00
Male	**1.08 (1.01, 1.16)**	**1.46 (1.27, 1.68)**	**1.18 (1.05, 1.32)**	**1.10 (1.02, 1.19)**	1.02 (0.87,1.20)	**1.11 (1.03, 1.20)**
Race/Ethnicity						
Non-Hispanic White	1.00	1.00	1.00	1.00	1.00	1.00
Non-Hispanic Black	1.07 (0.94, 1.22)	1.08 (0.88, 1.33)	**0.74 (0.61, 0.91)**	**0.54 (0.48, 0.61)**	**0.69 (0.50, 0.96)**	0.92 (0.82, 1.03)
Hispanic	**0.85 (0.77, 0.92)**	**0.77 (0.66, 0.89)**	0.87 (0.73, 1.05)	**0.69 (0.62, 0.78)**	**0.63 (0.54, 0.74)**	1.10 (0.98, 1.24)
Non-Hispanic Other ^c^	**0.78 (0.66, 0.92)**	**0.72 (0.63, 0.82)**	0.81 (0.64, 1.02)	0.89 (0.79, 1.01)	**0.81 (0.66, 0.98)**	0.96 (0.84, 1.10)
Cigarette Smoking Status						
Never	1.00	1.00	1.00	1.00	1.00	1.00
Current	**1.21 (1.01, 1.44)**	**1.59 (1.30, 1.94)**	0.88 (0.68, 1.13)	1.07 (0.96, 1.31)	0.99 (0.71, 1.4)	1.07 (0.83, 1.39)
Former	1.18 (0.99, 1.42)	**1.27 (1.03, 1.57)**	0.99 (0.76, 1.29)	**1.2 (1.04, 1.31)**	1.11 (0.80, 1.55)	1.17 (0.89, 1.54)

^a^ PATH restricted file received disclosure to publish: 2 March 2020. United States Department of Health and Human Services. National Institutes of Health. National Institute on Drug Abuse, and United States Department of Health and Human Services. Food and Drug Administration. Center for Tobacco Products. Population Assessment of Tobacco and Health (PATH) Study (United States) Restricted-Use Files. ICPSR36231-v13.AnnArbor, MI: Inter-university Consortium for Political and Social Research (distributor), 24 June 2020. https://doi.org/10.3886/ICPSR36231.v13. ^b^ Significant hazard ratios are bolded ^c^ Non-Hispanic other includes Asian, multi-race, etc.
